# Importance of optimizing duration of adjuvant immune checkpoint inhibitor therapy to treat postoperative hepatocellular carcinoma after conversion therapy: a case report

**DOI:** 10.1093/jscr/rjad610

**Published:** 2023-11-11

**Authors:** Jian-Rong Li, Da-Long Yang, Jin-Ming Wang, Wei Tian, Wei Wei, Cheng-Piao Luo, Lu-Nan Qi, Liang Ma, Jian-Hong Zhong

**Affiliations:** Hepatobiliary Surgery Department, Guangxi Medical University Cancer Hospital, Nanning 530021, China; Hepatobiliary Surgery Department, Guangxi Medical University Cancer Hospital, Nanning 530021, China; Hepatobiliary Surgery Department, Guangxi Medical University Cancer Hospital, Nanning 530021, China; Hepatobiliary Surgery Department, Guangxi Medical University Cancer Hospital, Nanning 530021, China; Radiology Department, Guangxi Medical University Cancer Hospital, Nanning 530021, China; Pathology Department, Guangxi Medical University Cancer Hospital, Nanning 530021, China; Hepatobiliary Surgery Department, Guangxi Medical University Cancer Hospital, Nanning 530021, China; Hepatobiliary Surgery Department, Guangxi Medical University Cancer Hospital, Nanning 530021, China; Hepatobiliary Surgery Department, Guangxi Medical University Cancer Hospital, Nanning 530021, China; Key Laboratory of Early Prevention and Treatment for Regional High Frequency Tumor (Guangxi Medical University), Ministry of Education, Nanning 530021, China; Guangxi Key Laboratory of Early Prevention and Treatment for Regional High Frequency Tumor, Nanning 530021, China

**Keywords:** adjuvant, hepatocellular carcinoma, immune checkpoint inhibitor, treatment duration, tislelizumab

## Abstract

Patients with hepatocellular carcinoma at high risk of recurrence after hepatic resection or local ablation often undergo adjuvant immunotherapy with immune checkpoint inhibitors for 1 year in randomized controlled trials, but the appropriateness of this duration is controversial, especially given the risk of adverse events. Here we report the case of a 52-year-old Chinese man with initially unresectable multinodular recurrent hepatocellular carcinoma who underwent two cycles of transarterial chemoembolization, followed by hepatic resection and 24 months of adjuvant therapy with the PD-1 inhibitor tislelizumab. The patient achieved a recurrence-free survival time of 24 months, but he experienced elevated alpha fetoprotein, Grade 2 hypothyroidism and pruritus while on adjuvant therapy. This case highlights the need to optimize the duration of adjuvant immunotherapy after curative treatment for hepatocellular carcinoma in order to minimize risk of not only recurrence but also adverse events.

## Introduction

Approximately 50%–70% of patients with hepatocellular carcinoma (HCC) experience recurrence within 5 years after potentially curative hepatic resection or ablation [1, 2]. Therefore, many patients receive adjuvant therapy after these curative treatments in order to improve long-term survival [3], and patients judged to be at higher risk of recurrence should be offered adjuvant immune checkpoint inhibitor (ICI) therapy, based on the newest guidelines from the American Association for the Study of Liver Diseases [4]. Several studies have linked such therapy to significantly longer recurrence-free or overall survival [5–8].

The application of conversion therapy to patients with unresectable HCC is increasing, but whether adjuvant ICI therapy is appropriate for such patients remains unclear [9–11]. Some work has suggested that adjuvant therapy can improve survival of patients who underwent conversion therapy [12]. Also, unclear is the optimal duration of such therapy. Here we describe a Chinese man with initially unresectable HCC who underwent conversion therapy followed by adjuvant ICI therapy. The patient achieved a recurrence-free survival time of 24 months, but he experienced several adverse effects because of the adjuvant therapy. This case suggests that while adjuvant ICI therapy can be effective after conversion therapy, its duration needs to be optimized.

## Case presentation

**Figure 1:**
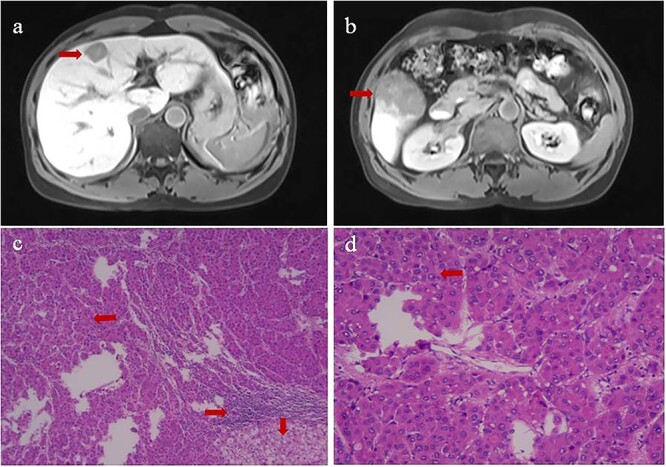
Contrast-enhanced magnetic resonance imaging at admission (upper row) and histopathology of primary tumors (lower row) of (**a**) the tumor at the junction of the left inner and upper right anterior lobes, and (**b**) the tumor in the lower right posterior lobe. Histopathology of the tumor in panels **c** and **d** showed tumor cells in Edmondson Grade II (leftward arrow), infiltration by macrophagocytes and lymphocytes (rightward arrow), and hepatocyte steatosis (downward arrows). Magnification, 40× in panel c or 100× in panel d.

A 52-year-old Chinese man was admitted to our hospital after physical examination revealed hepatic occupation that had persisted for the previous month. The patient had been chronically infected with hepatitis B virus for more than 20 years, and he had been diagnosed with hypertension 12 months previously, which he kept under control with nifedipine. Physical examination revealed no other unusual findings. His Eastern Cooperative Oncology Group score was 0, Child-Pugh score was 5, indocyanine green retention was 4.8% at 15 min, and level of alpha fetoprotein in serum exceeded 2000 ng/ml, for which no dilution test was performed (Supplementary Table 1). Contrast-enhanced magnetic resonance imaging revealed two massive nodular tumors in the liver, one at the junction of the left inner and upper right anterior lobes, and the other in the lower right posterior lobe (Fig. 1a and b).

The patient was diagnosed with HCC in Barcelona Clinic Liver Cancer Stage B. At 18 days after admission, the patient underwent hepatic resection of the right and middle lobes as well as cholecystectomy. Surgery proceeded uneventfully, and the patient recovered well. Postoperative histopathology confirmed that the two tumors were HCC and indicated an Edmondson grade of II (Fig. 1c and d). We detected one instance of microvascular invasion and one satellite nodule of Edmondson Grade II. Tumors expressed CK7 and glypican-3, but not CK19, based on immunohistochemistry. Surrounding liver tissue showed chronic changes associated with hepatitis, which we categorized as G1S1 on the inflammation grade (G) and fibrosis stage (S) [13].

We judged that our patient was at elevated risk of recurrence after resection because he had microvascular invasion, a satellite nodule, and multinodular tumors, so we administered one cycle of adjuvant transarterial embolization at 1 month after surgery in accordance with routine practices at our medical center [14, 15] and with Chinese HCC guidelines [16]. The patient was tested monthly for alpha-fetoprotein in serum, and he underwent contrast-enhanced computed tomography or magnetic resonance imaging every 3 months. He was also given antiviral therapy in accordance with Chinese guidelines [16]; we administered tenofovir because it may be more effective than entecavir [17]. The patient was discharged 1 week after surgery.

**Figure 2:**
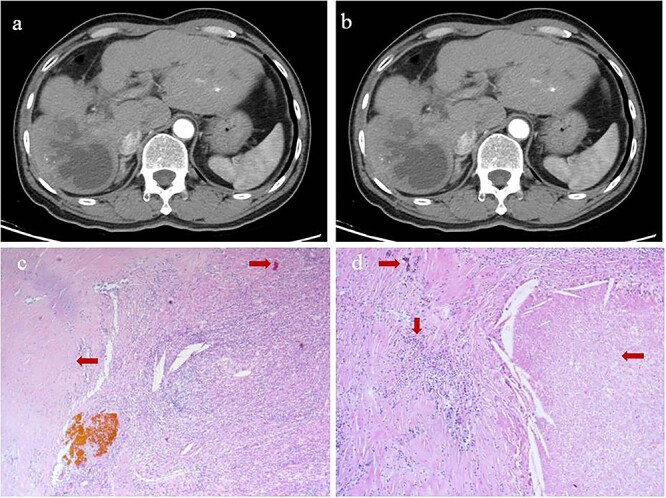
Intrahepatic recurrence and response to TACE. (**a**, **b**) Contrast-enhanced computed tomography in the (**a**) arterial phase or (**b**) venous phase after two cycles of TACE, showing PR of the recurrent tumor liver. (**c**, **d**) Histopathology of recurrent tumor after TACE at magnifications of (**c**) 40× or (**d**) 100×, showing necrosis of more than 90% of the tumor, proliferative fibrous tissue (leftward arrows), blue embolic agent in vasculature (rightward arrows), multinucleated giant cells, and abundant infiltration by lymphocytes (downward arrow).

At 24 months after his admission with hepatic occupation, contrast-enhanced computed tomography detected intrahepatic recurrence, which was confirmed by magnetic resonance imaging. The patient was admitted again and given two cycles of transarterial chemoembolization (TACE), which achieved a partial response (PR) based on the Response Evaluation Criteria for Solid Tumors [18] (Fig. 2a and b). At 16 weeks after his second admission, the patient underwent right hemihepatectomy. Baseline variables before surgery are described in Supplementary Table 1. The surgery proceeded smoothly, and the patient recovered well. Postoperative histopathology revealed necrosis in more than 90% of the tumor, whereas no nodular cirrhosis, satellite nodules, or tumor emboli were observed. Instead, necrotic and proliferative fibrous tissue with multinucleated giant cells was observed, with abundant infiltration by lymphocytes and focal aggregation of neutrophils (Fig. 2c and d). Surrounding liver tissue showed chronic hepatitis changes (G2S2 on the inflammation and fibrosis scale) as well as hyperplasia of the small bile duct.

**Figure 3:**
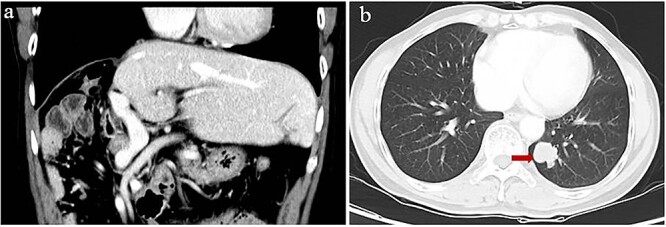
Computed tomography of lung metastasis after 2 years of adjuvant ICI. (**a**) Imaging at the venous stage did not reveal obvious lesions in the residual liver. (**b**) Imaging of the left lower lung revealed a single tumor (arrow).

Starting 1 month after repeat surgery, the patient received 200 mg of the ICI tislelizumab (BGB-A317; BeiGene, Beijing, China) once every 3 weeks via intravenous delivery lasting 60 min, in accordance with the manufacturer’s recommended dosing schedule. From 8 weeks on this therapy onwards, the level of alpha-fetoprotein in serum progressively increased. After 74 weeks on this therapy (21 treatments), the patient was diagnosed with Grade 2 hypothyroidism based on levels of thyroid hormone in serum and with Grade 2 pruritus based on signs and symptoms. Hypothyroidism was not treated because there were no obvious symptoms. However, he showed no signs of intra- or extrahepatic metastases in the entire body, abdomen, thoracic cavity, or brain based on magnetic resonance imaging or on contrast-enhanced, positron-emission, or bone-emission computed tomography. After 24 weeks on tislelizumab, a single metastasis was detected in the lung (Fig. 3). The patient was subjected to eight cycles of stereotactic body radiation therapy, and the level of alpha-fetoprotein in serum 3 weeks later was 275 ng/ml. Intensive follow-up continues.

## Discussion

Here we present a patient with initially unresectable HCC who underwent hepatic resection after conversion therapy with two cycles of TACE, and then received 24 months of adjuvant therapy with the PD-1 inhibitor tislelizumab. The patient achieved a recurrence-free survival time of 24 months, but he experienced Grade 2 hypothyroidism and pruritus during adjuvant therapy. Immune-related adverse events are common in adjuvant immunotherapy [6, 19, 20]. Therefore, it is very important to optimize the duration of adjuvant ICI therapy that limits the occurrence of chronic immune-related adverse events without compromising survival outcomes.

There is no consensus on the duration of adjuvant ICI therapy after curative treatment for HCC, and current clinical trials tend to administer the therapy for 1 year [5, 21–23]. One year may be too long, because several studies suggest that the efficacy of ICIs does not last long: median progression-free survival time was only 2–8 months in various trials involving patients who received first-line ICIs on their own [24–26] or with tyrosine kinase inhibitors [27–30], or who received second-line ICIs with or without tyrosine kinase inhibitors [31–35] (Supplementary Table 2). Some non-randomized studies have administered adjuvant ICIs after hepatic resection for only 4–6 months, which was sufficient to achieve a significant reduction in risk of recurrence [6, 8].

Another reason to optimize the duration of adjuvant ICI therapy is that such inhibitors may increase risk of chronic adverse events related to the immune system [19, 20]. Monitoring and managing persistent toxic effects can be particularly cumbersome in HCC patients because such patients normally would not require close management because their cancer has been cured.

**Figure 4:**
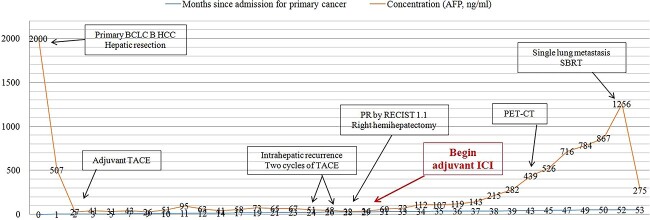
Concentration of alpha-fetoprotein in the patient’s serum over the course of clinical interventions. BCLC, Barcelona Clinic Liver Cancer; PET-CT, positron-emission tomography/computed tomography; RECIST, Response Evaluation Criteria in Solid Tumors; SBRT, stereotactic body radiation therapy.

Our patient showed a recurrence-free survival time of 24 months after the first surgery (Fig. 4), slightly longer than the 20.3 months reported for patients receiving adjuvant hepatic artery infusion chemotherapy with 5-fluorouracil and oxaliplatin [36]. Our patient may also achieve recurrence-free survival of 24 months after repeat surgery: after nearly 24 months on adjuvant tislelizumab, no lesions were found on imaging even though the level of alpha-fetoprotein in serum remained slightly elevated. Such recurrence-free survive would be similar to the 25.2 months reported for patients at our medical center who received adjuvant ICIs with or without tyrosine kinase inhibitors after curative surgery [6].

The alpha-fetoprotein level in our patient’s serum began to rise rapidly after 8 months of adjuvant ICI treatment; after 16 months of treatment, lung metastasis was detected. Our experience highlights the usefulness of elevated alpha-fetoprotein as a marker of tumor recurrence, metastasis, or *de novo* growth [11]. Moreover, this case report highlights the need for non-inferiority clinical trials to explore shorter adjuvant ICI therapy to maximize benefit while minimizing risks. Lastly, our case justifies further studies into the effects of adjuvant ICIs on recurrence in HCC patients after curative treatment.

There were some limitations in this study. First, there was no needle biopsy for the recurrent tumor, and the tumor cells were almost necrotic after conversion therapy, so immunohistochemical analysis could not be performed. Therefore, significant tumor heterogeneity between the primary and the recurrent HCC cannot be ruled out because alpha-fetoprotein was not significantly elevated at the first HCC recurrence. We did not genetically profile the primary and recurrent tumors, which future work should do.

### Patient perspective

The recurrence and metastasis of my HCC have burdened me and my family, and we thank my doctors for formulating a variety of effective treatments for me. We continued adjuvant immunotherapy beyond 1 year because the level of alpha-fetoprotein in my serum continued to rise, yet various types of imaging failed to detect any lesions. The prolonged immunotherapy led to immune-related adverse events. After consulting with my physician and reviewing the relevant medical literature myself, I see there is no consensus about the duration of effective adjuvant therapy.

## Ethics statement

Ethical approval was provided for this study (LW2023130). The patient provided his written informed consent to participate in this study. Written informed consent was obtained from the individual for the publication of any potentially identifiable images or data included in this article.

## Supplementary Material

supplementary_table_rjad610

## Data Availability

The raw data supporting the conclusions of this article will be made available by the authors, without undue reservation.

## References

[1] Zhong JH, Xing BC, Zhang WG, et al. Repeat hepatic resection versus radiofrequency ablation for recurrent hepatocellular carcinoma: retrospective multicentre study. Br J Surg 2022;109:71–8. 10.1093/bjs/znab340 34643677

[2] Yuan BH, Zhu YK, Zou XM, et al. Repeat hepatic resection versus percutaneous ablation for the treatment of recurrent hepatocellular carcinoma: meta-analysis. BJS Open 2022;6:zrac036. 35482024 10.1093/bjsopen/zrac036 PMC9048940

[3] Zhang EL . Editorial: immunomodulatory factors, conversion, and postoperative adjuvant therapy for hepatobiliary tumors based on immunotherapy. Front Immunol 2023;14:1218845. 37287965 10.3389/fimmu.2023.1218845 PMC10242167

[4] Singal AG, Llovet JM, Yarchoan M, et al. AASLD practice guidance on prevention, diagnosis, and treatment of hepatocellular carcinoma. Hepatology 2023. 10.1097/HEP.0000000000000466. PMC10663390 37199193

[5] Qin S, Chen M, et al. IMbrave050 investigators. Atezolizumab plus bevacizumab versus active surveillance in patients with resected or ablated high-risk hepatocellular carcinoma (IMbrave050): a randomised, open-label, multicentre, phase 3 trial. Lancet. 2023;S0140-6736(23)01796-8. 10.1016/S0140-6736(23)01796-8. 37871608

[6] Li L, Wu PS, Liang XM, et al. Adjuvant immune checkpoint inhibitors associated with higher recurrence-free survival in postoperative hepatocellular carcinoma (PREVENT): a prospective, multicentric cohort study. J Gastroenterol 2023;58:1043–54. 37452107 10.1007/s00535-023-02018-2

[7] Li J, Wang WQ, Zhu RH, et al. Postoperative adjuvant tyrosine kinase inhibitors combined with anti-PD-1 antibodies improves surgical outcomes for hepatocellular carcinoma with high-risk recurrent factors. Front Immunol 2023;14:1202039. 37359534 10.3389/fimmu.2023.1202039 PMC10285103

[8] Chen W, Hu S, Liu Z, et al. Adjuvant anti-PD-1 antibody for hepatocellular carcinoma with high recurrence risks after hepatectomy. Hepatol Int 2023;17:406–16. 36645648 10.1007/s12072-022-10478-6

[9] Sun HC, Zhou J, Wang Z, et al. Chinese expert consensus on conversion therapy for hepatocellular carcinoma (2021 edition). Hepatobiliary Surg Nutr 2022;11:227–52. 35464283 10.21037/hbsn-21-328 PMC9023831

[10] Lan XB, Papatheodoridis G, Teng YX, et al. The upward trend in the immunotherapy utilization for hepatobiliary cancers. Hepatobiliary Surg Nutr 2021;10:692–5. 34760976 10.21037/hbsn-21-342 PMC8527408

[11] Chen K, Luo CP, Ge DX, et al. Case report: conversion therapy to permit resection of initially unresectable hepatocellular carcinoma. Front Oncol 2022;12:946693. 36276151 10.3389/fonc.2022.946693 PMC9583878

[12] Pan Y, Yuan Z, Wang J, et al. Survival benefit and impact of adjuvant therapies following FOLFOX-HAIC-based conversion therapy with unresectable hepatocellular carcinoma: a retrospective cohort study. J Cancer Res Clin Oncol 2023;149:14761–74. 10.1007/s00432-00023-05243-00437. PMC11797165 37589925

[13] Goodman ZD . Grading and staging systems for inflammation and fibrosis in chronic liver diseases. J Hepatol 2007;47:598–607. 17692984 10.1016/j.jhep.2007.07.006

[14] Qi YP, Zhong JH, Liang ZY, et al. Adjuvant transarterial chemoembolization for patients with hepatocellular carcinoma involving microvascular invasion. Am J Surg 2019;217:739–44. 30103903 10.1016/j.amjsurg.2018.07.054

[15] Zhong JH, Li LQ. Postoperative adjuvant transarterial chemoembolization for participants with hepatocellular carcinoma: a meta-analysis. Hepatol Res 2010;40:943–53. 20887328 10.1111/j.1872-034X.2010.00710.x

[16] Zhou J, Sun H, Wang Z, et al. Guidelines for the diagnosis and treatment of hepatocellular carcinoma (2019 edition). Liver Cancer 2020;9:682–720. 33442540 10.1159/000509424 PMC7768108

[17] Yuan BH, Li RH, Huo RR, et al. Lower risk of hepatocellular carcinoma with tenofovir than entecavir treatment in subsets of chronic hepatitis B patients: an updated meta-analysis. J Gastroenterol Hepatol 2022;37:782–94. 35080052 10.1111/jgh.15783

[18] Eisenhauer EA, Therasse P, Bogaerts J, et al. New response evaluation criteria in solid tumours: revised RECIST guideline (version 1.1). Eur J Cancer 2009;45:228–47. 19097774 10.1016/j.ejca.2008.10.026

[19] Patrinely JR Jr, Johnson R, Lawless AR, et al. Chronic immune-related adverse events following adjuvant anti-PD-1 therapy for high-risk resected melanoma. JAMA Oncol 2021;7:744–8. 33764387 10.1001/jamaoncol.2021.0051 PMC7995124

[20] Goodman RS, Lawless A, Woodford R, et al. Extended follow-up of chronic immune-related adverse events following adjuvant anti-PD-1 therapy for high-risk resected melanoma. JAMA Netw Open 2023;6:e2327145. 37535354 10.1001/jamanetworkopen.2023.27145 PMC10401300

[21] Vogel A, Zhu AX, Cheng AL, et al. 1017TiP KEYNOTE-937 trial in progress: adjuvant pembrolizumab in patients with hepatocellular carcinoma (HCC) and complete radiologic response after surgical resection or local ablation. Ann Oncol 2020;31:S703.

[22] Jimenez Exposito MJ, Akce M, Alvarez JLM, et al. 209TiP - CA209-9DX: phase III, randomized, double-blind study of adjuvant nivolumab vs placebo for patients with hepatocellular carcinoma (HCC) at high risk of recurrence after curative resection or ablation. Ann Oncol 2018;29:ix65.

[23] Knox J, Cheng A, Cleary S, et al. P-187 - a phase 3 study of durvalumab with or without bevacizumab as adjuvant therapy in patients with hepatocellular carcinoma (HCC) who are at high risk of recurrence after curative hepatic resection. Ann Oncol 2019;30:iv51.

[24] El-Khoueiry AB, Sangro B, Yau T, et al. Nivolumab in patients with advanced hepatocellular carcinoma (CheckMate 040): an open-label, non-comparative, phase 1/2 dose escalation and expansion trial. Lancet 2017;389:2492–502. 28434648 10.1016/S0140-6736(17)31046-2 PMC7539326

[25] Yau T, Park JW, Finn RS, et al. Nivolumab versus sorafenib in advanced hepatocellular carcinoma (CheckMate 459): a randomised, multicentre, open-label, phase 3 trial. Lancet Oncol 2022;23:77–90. 34914889 10.1016/S1470-2045(21)00604-5

[26] Qin S, Kudo M, Meyer T, et al. LBA36 final analysis of RATIONALE-301: randomized, phase III study of tislelizumab versus sorafenib as first-line treatment for unresectable hepatocellular carcinoma. Ann Oncol 2022;33:S1402–3.

[27] Qin S, Chan SL, Gu S, et al. Camrelizumab plus rivoceranib versus sorafenib as first-line therapy for unresectable hepatocellular carcinoma (CARES-310): a randomised, open-label, international phase 3 study. Lancet 2023;402:1133–46. 37499670 10.1016/S0140-6736(23)00961-3

[28] Ren Z, Xu J, Bai Y, et al. Sintilimab plus a bevacizumab biosimilar (IBI305) versus sorafenib in unresectable hepatocellular carcinoma (ORIENT-32): a randomised, open-label, phase 2-3 study. Lancet Oncol 2021;22:977–90. 34143971 10.1016/S1470-2045(21)00252-7

[29] Finn RS, Qin S, Ikeda M, et al. Atezolizumab plus bevacizumab in unresectable hepatocellular carcinoma. N Engl J Med 2020;382:1894–905. 32402160 10.1056/NEJMoa1915745

[30] Finn RS, Kudo M, Merle P, et al. LBA34 primary results from the phase III LEAP-002 study: Lenvatinib plus pembrolizumab versus lenvatinib as first-line (1L) therapy for advanced hepatocellular carcinoma (aHCC). Ann Oncol 2022;33:S1401.

[31] Xu J, Shen J, Gu S, et al. Camrelizumab in combination with Apatinib in patients with advanced hepatocellular carcinoma (RESCUE): a nonrandomized, open-label, phase II trial. Clin Cancer Res 2021;27:1003–11. 33087333 10.1158/1078-0432.CCR-20-2571

[32] Zhu AX, Finn RS, Edeline J, et al. Pembrolizumab in patients with advanced hepatocellular carcinoma previously treated with sorafenib (KEYNOTE-224): a non-randomised, open-label phase 2 trial. Lancet Oncol 2018;19:940–52. 29875066 10.1016/S1470-2045(18)30351-6

[33] Finn RS, Ryoo BY, Merle P, et al. Pembrolizumab as second-line therapy in patients with advanced hepatocellular carcinoma in KEYNOTE-240: a randomized, double-blind, phase III trial. J Clin Oncol 2020;38:193–202. 31790344 10.1200/JCO.19.01307

[34] Qin S, Chen Z, Fang W, et al. Pembrolizumab versus placebo as second-line therapy in patients from Asia with advanced hepatocellular carcinoma: a randomized, double-blind, phase III trial. J Clin Oncol 2023;41:1434–43. 36455168 10.1200/JCO.22.00620 PMC9995104

[35] Qin S, Ren Z, Meng Z, et al. Camrelizumab in patients with previously treated advanced hepatocellular carcinoma: a multicentre, open-label, parallel-group, randomised, phase 2 trial. Lancet Oncol 2020;21:571–80. 32112738 10.1016/S1470-2045(20)30011-5

[36] Li SH, Mei J, Cheng Y, et al. Postoperative adjuvant hepatic arterial infusion chemotherapy with FOLFOX in hepatocellular carcinoma with microvascular invasion: a multicenter, phase III, randomized study. J Clin Oncol 2023;41:1898–908. 36525610 10.1200/JCO.22.01142 PMC10082249

