# Supplementary material for: Importance of optimizing duration of adjuvant immune checkpoint inhibitor therapy to treat postoperative hepatocellular carcinoma after conversion therapy: a case report
Source: J Surg Case Rep. 2023 Nov 11;2023(11):rjad610. doi: 10.1093/jscr/rjad610 (PMC10641292; doi:10.1093/jscr/rjad610)
Supplement: supplementary_table_rjad610 [file supplementary_table_rjad610.docx]

**Supplementary table 1.** Baseline clinical characteristics of the patient.

| **Characteristic** | **Before first hepatectomy (March 2019)** | **Before repeat hepatectomy (August 2021)** |
| --- | --- | --- |
| HBsAg | 332.2 | 103.9 |
| HBV DNA, IU/ml | 639 | 34.7 |
| Alpha-fetoprotein, ng/ml | >2000 | 31.6 |
| PIVKA-II, ng/ml | NA | 19.9 |
| Platelet, 10^9^/L | 320 | 222 |
| Total bilirubin, umol/L | 5.1 | 19.3 |
| Albumin, g/L | 35.4 | 32.3 |
| Alanine aminotransferase, U/L | 22 | 65 |
| Prothrombin time, s | 15.0 | 13.6 |
| Tumor dimensions, cm | 5.0 x 4.5 x 3.0 and 2.0 x 2.0 x 1.6 | 9.5 x 6.5 x 6.0 |
| Tumor number | 2 | Multiple |
| Macrovascular invasion | Absent | Absent |
| BCLC stage | B | B |
| Microvascular invasion | Present | Absent |
| Tumor satellites | Present | Absent |

BCLC, Barcelona Clinic Liver Cancer; HBsAg, hepatitis B virus surface antigen; HBV, hepatitis B virus; PIVKA-II, protein induced by vitamin K absence or antagonist-II.

**Supplementary table 2.**  Summary of data from published trials of immune checkpoint inhibitors in hepatocellular carcinoma.

| **Trial or study** | **Treatment and sample size** | **Phase** | **Median PFS time, months** | **HR (95%CI)** |
| --- | --- | --- | --- | --- |
| **First-line** |  |  |  |  |
| CheckMate 040^24^ | Nivolumab dose-expansion (*n*=214) *vs* dose-escalation phase (*n*=48) | I/II | 4.0 *vs* 3.4 | - |
| CheckMate 459^25^ | Nivolumab (*n*=371) *vs* sorafenib (*n*=372) | III | 3.8 *vs* 3.7 | 0.93 (0.79-1.10) |
| RATIONALE-301^26^ | Tislelizumab (n=342) *vs* sorafenib (n=332) | Ⅲ | 2.2 *vs* 3.6 | 1.10 (0.92-1.33) |
| LEAP-002^30^ | Lenvatinib plus pembrolizumab (n=395) vs lenvatinib (n=399) | Ⅲ | 8.2 *vs* 8.0 | 0.83 (0.71-0.98) |
| IMbrave 150^29^ | Atezolizumab plus bevacizumab (*n*=336) *vs* sorafenib (*n*=165) | Ⅲ | 6.8 *vs* 4.3 | 0.59 (0.47-0.76) |
| ORIENT-32^28^ | Sintilimab plus bevacizumab (*n*=380) *vs* sorafenib (*n*=191) | Ⅲ | 4.6 *vs* 2.8 | 0.56 (0.46-0.70) |
| CARES-310^27^ | Camrelizumab plus apatinib (n=272) *vs* sorafenib (n=271) | Ⅲ | 5.6 *vs* 3.7 | 0.52 (0.41-0.65) |
| **Second-line** |  |  |  |  |
| RESCUE^31^ | Camrelizumab plus apatinib (*n*=120) | II | 5.5 | - |
| Qin et al. 2020^35^ | Camrelizumab (*n*=217) | II | 2.1 | - |
| KEYNOTE-224^32^ | Pembrolizumab (*n*=104) | II | 4.9 | - |
| KEYNOTE-240^33^ | Pembrolizumab (*n*=278) *vs* placebo (*n*=135) | III | 3.0 *vs* 2.8 | 0.72 (0.57-0.90) |
| KEYNOTE-394^34^ | Pembrolizumab (*n*=300) *vs* placebo (*n*=153) | III | 2.6 *vs* 2.3 | 0.74 (0.60-0.92) |
| **Adjuvant** |  |  |  |  |
| Imbrave 050^5^ | Atezolizumab plus bevacizumab (*n*=334) *vs* active surveillance (*n*=334) | III | NR *vs* NR^*^ | 0.72 (0.56-0.93) |
| PREVENT^6^ | ICI with or without TKI (n=85) *vs* empty control (n=432) | Prospective | 25.2 *vs* 16.1^*^ | 0.64 (0.46-0.88) |
| Chen et al. 2023^8^ | ICI (n=47) *vs* empty control (n=47) | Retrospective | 17.7 *vs* 5.7^*^ | 0.48 (0.28-0.83) |
| Li et al. 2023^7^ | ICI plus TKI (n=47) *vs* empty control (n=47) | Retrospective | NR *vs* 9.8^*^ | 0.33 (0.18-0.60) |

*Median recurrence-free survival time (months). CI, confidence interval; HR, hazard ratio; ICI, immune checkpoint inhibitor; NR, not reached; PFS, progression-free survival; TKI, tyrosine kinase inhibitor.
